# The complete mitochondrial genome of *Thor amboinensis* (Hippolytidae, Decapoda)

**DOI:** 10.1080/23802359.2020.1797591

**Published:** 2020-07-30

**Authors:** Yan Wang, Ling Zeng, Jing Wen, Xuyan Li, Yafen Huang, Yulin Sun, Juan Zhao

**Affiliations:** a Department of Scientific Research, Lingnan Normal University, Zhanjiang, China; b Department of Chemistry, Lingnan Normal University, Zhanjiang, China; c Department of Biology, Lingnan Normal University, Zhanjiang, China

**Keywords:** *Thor amboinensis*, mitochondrial genome, phylogenetic analysis, Hippolytidae

## Abstract

The complete mitochondrial genome of *Thor amboinensis* was obtained and described in this study. This complete mitochondrial genome is 15,553 bp in length and consists of 13 protein-coding genes, 2 ribosomal RNA genes, and 22 transfer RNA genes. Twenty-two genes were encoded by the heavy strand. The overall base composition of the heavy-strand was 36.09% A, 12.36% G, 14.54% C, and 37.01% T, with a high G + C content of 26.90%. The phylogenetic analysis suggested that *T. amboinensis* was closest to *Lebbeus groenlandicus*. The newly described mitochondrial genome may provide valuable data for phylogenetic analysis for Hippolytidae.

The trade of marine ornamental species has been increased rapidly (Balaji et al. 2009). *Thor amboinensis*, also named sexy shrimp, has bright color. *Thor amboinensis* usually raises their tail and curious body when walking (Calado et al. 2007). This fascinating behavior and their beautiful colors of *T. amboinensis* make it popular on the ornamental industry (Debelius 2001; Calado et al. 2007)*. Thor amboinensis* is naturally distributed in a circum-tropical sea area, usually living free or associated with corals and anemones (Debelius 2001). *Thor amboinensis* first matures into male and then changes into female later in life (Baeza and Piantoni 2010). Complete larval development of *T. amboinensis*, including eight zoeal stages and one decapodid, was identified (Bartilotti et al. 2016). Unfortunately, only a partial cox1 DNA sequences of *T. amboinensis* could be found in GenBank. Lack of genetic resources has hindered conservation and utilization of *T. amboinensis.* In this study, the complete mitochondrial genome of *T. amboinensis* was reported, which provides genomic data for phylogenetic and evolutionary investigations of *Thor* and Hippolytidae.

The specimen was collected from Shenzhen, Guangdong province, China (N22°35′, E114°31′) and deposited in the Zoological Herbarium, Lingnan Normal University (acc. number SC20200415-9). The muscle of *T. amboinensis* was fixed in 100% ethanol and stored at –20 °C. Approximately, 30 mg of muscle tissue was used for mitochondrial DNA (mtDNA) extraction with TIANamp Marine Animals DNA Kit (Tiangen, Beijing, China) according to the manufacturer’s specification. MtDNA was sequenced using the Illumina Hiseq Sequencing System (Illumina Inc., San Diego, CA). The clean data were acquired and assembled by the SPAdes and PRICE (Bankevich et al. 2012). BLAST (http://www.ncbi.nlm.nih.gov/BLAST/), ORFs finder (https://www.ncbi.nlm.nih.gov/orffinder/), and MITO (Bernt et al. 2013) were used to identify and annotate protein-coding genes (PCGs). tRNAscan-SE 2.0 (Lowe and Chan 2016), and MITO (Bernt et al. 2013) were used to identify tRNA genes. A phylogenetic tree was constructed using MEGA 6.0 software.

The mitochondrial genome of *T. amboinensis* is 15,553 bp in length (GenBank accession number: MT671809) and contains a typical set of 13 PCGs, 22 tRNA, and two rRNA genes. The heavy strand consists of 36.09% A, 12.36% G, 14.54% C, and 37.01% T, with a high G + C content of 26.90%. Of the 37 genes, 4 PCGs, 8 tRNA, and 2 rRNA were encoded by the light strand, and 23 were encoded by the heavy strand. Six PCGs were initiated by ATG. Cox1, ND1, and ND3 were initiated by ATA. ND6 and cytb were started with ATT and ND2 was started with ATC. Seven PCGs were terminated with the TAA as stop codon, ND2, cox1, and ND4 were terminated with the TAG. ND4 L was finished with the TGT, while cox2 and cytb were terminated with the T–. Two rRNA genes, 12S rRNA and 16S rRNA were 796 bp and 1084 bp in size, respectively.

Based on the complete 13 concatenated PCGs from 27 shrimps from GenBank database, a phylogenetic tree was constructed by maximum likelihood (ML) method (Figure 1). It was demonstrated that *T. amboinensis* was clustered with *Lebbeus groenlandicus*, which suggested that the *T. amboinensis* is closely related to *L. groenlandicus*. Both of them belong to Hippolytidae family. This newly reported genome of *T. amboinensis* will contribute to future phylogenetic studies and population genetic analyses for *T. amboinensis*.

**Figure 1.**
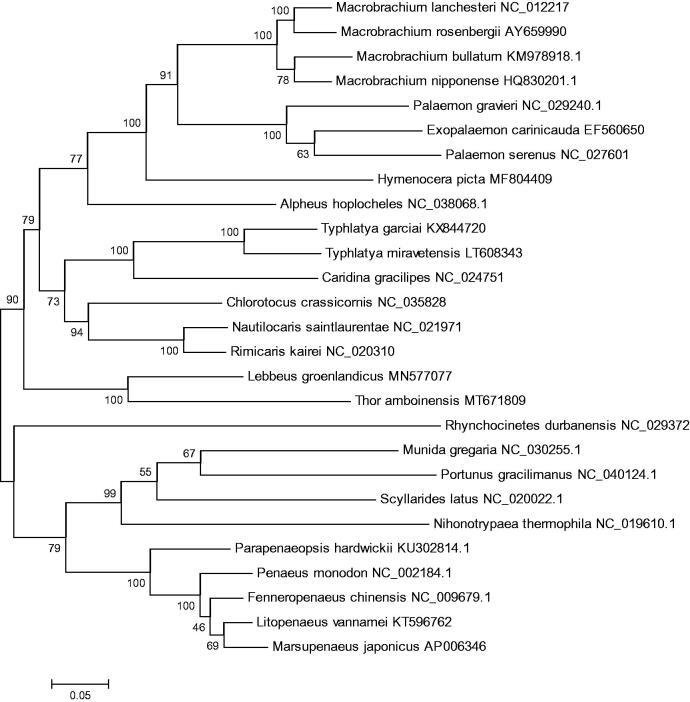
Phylogenetic tree of *T. amboinensis* and related species based on maximum likelihood (ML) method.

## Data Availability

The data that support the findings of this study are openly available in NCBI at https://www.ncbi.nlm.nih.gov/, reference number MT671809.
